# Influence of Temperature on the Mechanical Properties and Reactive Behavior of Al-PTFE under Quasi-Static Compression

**DOI:** 10.3390/polym10010056

**Published:** 2018-01-10

**Authors:** Huai-Xi Wang, Xiang Fang, Bin Feng, Zhen-Ru Gao, Shuang-Zhang Wu, Yu-Chun Li

**Affiliations:** 1 College of Field Engineering, PLA Army Engineering University, Nanjing 210007, China; 15251853685@163.com (H.-X.W.); gygzr@sina.com (Z.-R.G.); shsnake@163.com (S.-Z.W.); 2 China Huayin Ordnance Test Center, Huayin 714200, China; fengbinplaust@gmail.com

**Keywords:** Al-PTFE, quasi-static compression, mechanical response, reactive behavior, temperature

## Abstract

Al-PTFE (aluminum-polytetrafluoroethylene) is a typical kind of Reactive Material (RM), which has a variety of potential applications in weapon systems. In this paper, quasi-static compression experiments were carried out for a pressed and sintered mixture of Al and PTFE powders using a microcomputer-controlled electronic universal testing machine. The results show that both the mechanical property and reactive behavior of Al-PTFE are strongly temperature-dependent. The material undergoes a brittle-ductile transition associated with a temperature-induced crystalline phase transformation of the PTFE matrix. At low temperatures (−18, 0, and 16 °C), samples of Al-PTFE failed with shear crack and no reaction was observed. As the temperature increased (22, 35, and 80 °C), Al-PTFE exhibited a high toughness and violent reaction occurred in all of the tested samples. Scanning electron microscope observations showed different fracture mechanisms of the PTFE matrix and the increase in toughness was due to the formation of PTFE fibrils which could dissipate energy and bridge crack plane during plastic deformation.

## 1. Introduction

Reactive Material (RM), which consists of at least two non-explosive solid components, can release a great amount of energy and make effective damage upon impacting with targets. Al-PTFE (aluminum-polytetrafluoroethylene) is regarded as a typical kind of RM, for which the synthesis process was first reported by Joshi [1]. When the composite of Al-PTFE is mixed at the stoichiometric ratio of 26.5:73.5 by weight, the theoretical energy released could reach up to 8.68 kJ/g [2]. Due to its high energy density, Al-PTFE has received much attention in the past decades.

To ensure its safety and reliability in the process of manufacture and application, it is important to have a thorough understanding of the mechanical response of Al-PTFE under different conditions. Raftenberg et al. [3] carried out quasi-static compression tests and high-rate split Hopkinson bar experiments for Al-PTFE. The data measured were used to determine the parameters for the Johnson-Cook model and the consistency was examined between the simulation and experiment in terms of specimen shape versus time. Ge et al. [4] studied the mechanical behavior of Al-PTFE with a numerical method at the microscale. The models were based on real microstructures of Al-PTFE and the microstructural parameters, such as the geometry, distribution, and sizes of Al particles, were taken into account. Cai et al. [5] found that the addition of W could effectively improve the strength of Al-PTFE. Subsequently, Xu et al. [6], Zhang et al. [7], Wang et al. [8], and Ge et al. [9] investigated the mechanical behavior of Al-W-PTFE by differing the W content and particle size. Although the influencing factors on the mechanical property of Al-PTFE have been extensively studied in the literature listed above, very little attention has been paid to the effect of temperature. 

Generally, Al-PTFE is thought to be inert under quasi-static compression. However, Feng et al. [10] reported a reaction phenomenon of Al-PTFE samples after a specific heat treatment procedure under quasi-static loading. Micrographs and finite element simulation of the sample in the critical initiation state suggested that the reaction was more like a mechanochemical process rather than a thermochemical one. In the following study [11], they investigated the influence of sintering temperature, equivalence ratio, and Al particle size on the reaction phenomenon under quasi-static compression and a crack-induced initiation mechanism was proposed, while the effect of temperature was not taken into account either.

**Figure 1:**
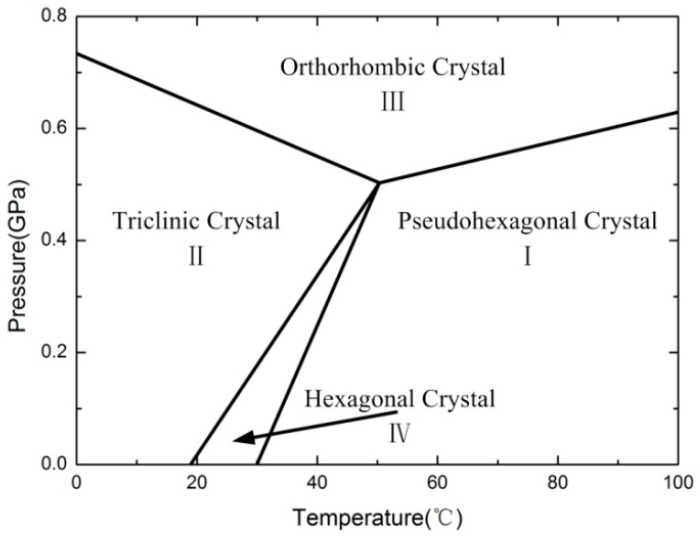
Crystalline phases of PTFE (polytetrafluoroethylene) as a function of temperature and pressure.

PTFE is a kind of semi-crystalline polymer in nature. The crystalline material is embedded in the amorphous regions and the crystalline regions are interconnected by amorphous chains [12]. The melting point (the first-order transition from a partly crystalline to a completely amorphous structure) is about 330 °C, which is much higher than that of the corresponding hydrocarbon polymer [13]. The glass transitions in the amorphous regions of PTFE are located at −97 and 127 °C [14,15,16], both of which are second-order transitions. In the crystalline regions there are four distinct crystalline phases depending on pressure and temperature [17,18], which are shown in Figure 1. The figure is plotted for illustrative purposes only and the pressure dependencies are not exactly correct. As can be seen from Figure 1, PTFE exhibits three crystalline phases at ambient pressure. The first crystalline phase transition between phases II and IV happens at 19 °C, which is an unraveling in the helical conformation from a well-ordered triclinic structure with 13 atoms/180° turn [19] to a partially ordered hexagonal structure with 15 atoms/180° turn [20]. Further rotational disordering and untwisting of the helices occurs at 30 °C, giving way to phase I to form a pseudohexagonal structure [21]. The first-order transitions between crystalline phases of PTFE are reversible and unique among polymers [13], and the temperatures that Al-PTFE is likely to encounter in application cover the two phase transition temperatures, so it is essential to develop an understanding of the effects of temperature-induced phase transitions on the properties of Al-PTFE over the normal range of operating temperatures.

In this paper, the mechanical properties and reactive behavior of Al-PTFE under quasi-static compression were investigated at temperatures between −18 and 80 °C, which were chosen to encompass the three crystalline phases of PTFE with transitions at 19 and 30 °C. The results are presented and described in detail, and the relationship between mechanical property and reaction phenomenon of Al-PTFE is built.

## 2. Materials and Experiments

### 2.1. Sample Preparation

**Figure 2:**
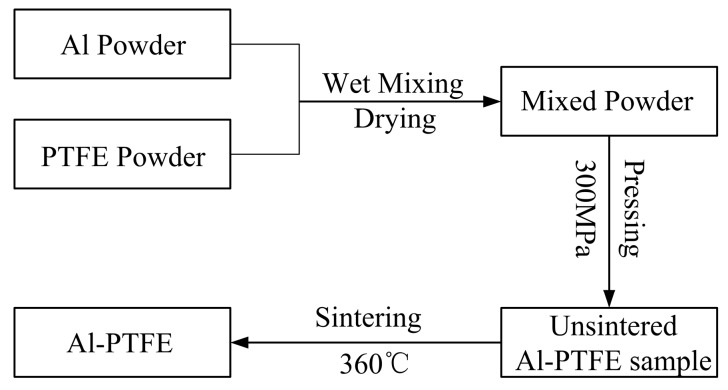
The preparation process of Al-PTFE samples.

Al (purity 99.5%, average particle size 1 μm, from Jin Tian, Luxi County, Hunan, China) and PTFE (purity 99%, average particle size 25 μm, from 3M, Shanghai, China) were mixed at the stoichiometric ratio of 26.5:73.5 by weight. The preparation process was based on the patent of Nielson et al. [22], which is shown in Figure 2, along with the main steps described below.

**Figure 3:**
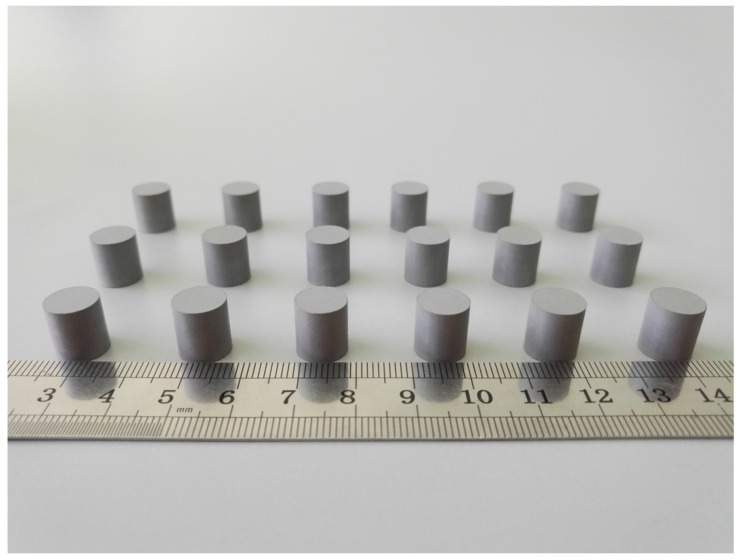
Samples of Al-PTFE for quasi-static compression tests.

Firstly, Al and PTFE powders were put into a beaker containing ethanol solution, and stirred by an electric blender for at least 20 min to ensure a thorough mixing. Then the mixed solution was dried at 60 °C for 48 h, to make the ethanol fully evaporate. Secondly, the dried mixture was pressed into samples with a size of ∅10 mm × 10 mm for quasi-static compression tests under a pressure of 300 MPa. To prevent the rebound of the sample, the pressure was held for a minimum of 1 min. Finally, the pressed samples were sintered in a vacuum oven. The oven was heated to the sintering temperature of 360 °C at a heating rate of 90 °C/h. Samples were soaked in the sintering temperature for 4 h and cooled to room temperature at a cooling rate of 50 °C/h. The sintering process was realized by a programmable temperature controller embedded in the oven. The samples after sintering are shown in Figure 3.

**Figure 4:**
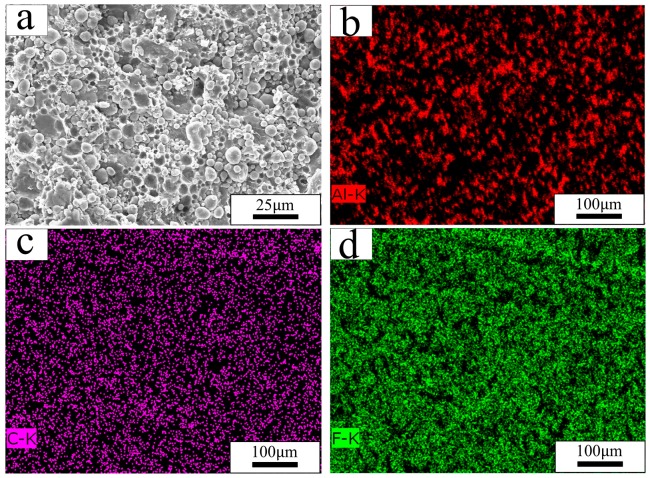
Internal SEM image and element distributions of the Al-PTFE sample: (**a**) SEM image; (**b**) Al element; (**c**) C element; (**d**) F element.

In order to examine the homogeneity of prepared material, a Hitachi S-4800 scanning electron microscope (SEM), from HITACHI, Tokyo, Japan, was used to observe the interior of the sample (Figure 4). The microvoids in Figure 4a were formed by Al particles, which were on the opposite side of the observation area. It is obvious that Al particles are uniformly distributed in PTFE matrix. The distributions of Al, C, and F (Figure 4b–d) further indicate that the Al and PTFE powders were homogeneously mixed through the preparation process outlined in this paper.

### 2.2. Quasi-Static Compression Tests

Quasi-static compression tests were carried out by a CMT5105 microcomputer-controlled electronic universal testing machine (MTS industry system (Chinese) Co. Ltd., Shenzhen, Guangdong, China) with a loading capacity of 100 kN, and the standard used was GB/T 1041-2008 (Plastics—Determination of compressive properties). The load was applied at a speed of 60 mm/min, corresponding to the nominal strain rate of 0.1/s. A digital video camera with a frame frequency of 40 Hz was used to record the reaction phenomenon that occurred in the compression process. Prior to the tests, samples were soaked at desired test temperature for at least 2 h to ensure thermal equilibrium. Due to the friction on contact surfaces, the ends of samples were retarded from moving outward during deformation. Therefore, petroleum jelly was used to lubricate both ends of the sample to alleviate the friction. Three samples were tested at each temperature to examine the reproducibility of experimental results.

According to the data collected by the testing machine, the engineering stress and engineering strain could be calculated by the following equations:

(1) $$\left\{ \begin{array}{l} \sigma_{e}\;=\;\frac{P}{A_{0}}\\ \varepsilon_{e}\;=\;\frac{h_{0}-h}{h_{0}} \end{array}\right.$$

where $\sigma_{e}$ and $\varepsilon_{e}$ are the engineering stress and engineering strain, respectively. P is the applied load and h is the instantaneous height of the sample during the compression test. $A_{0}$ and $h_{0}$ are the initial cross-sectional area and height of the sample, respectively. Based on the assumption of a constant sample volume during deformation, the true stress and true strain could be expressed as:

(2) $$\left\{ \begin{array}{l} \sigma_{t}\;=\;\frac{P}{A}\;=\;\frac{P}{A_{0}}(1-\varepsilon_{e})\\ \varepsilon_{t}\;=\;\ln\frac{h}{h_{0}}\;=\;\ln\frac{1}{1-\varepsilon_{e}} \end{array}\right.$$

where $\sigma_{t}$ and $\varepsilon_{t}$ are the true stress and true strain, respectively. A is the instantaneous cross-sectional area of the sample.

## 3. Results and Discussion

### 3.1. Mechanical Response

**Figure 5:**
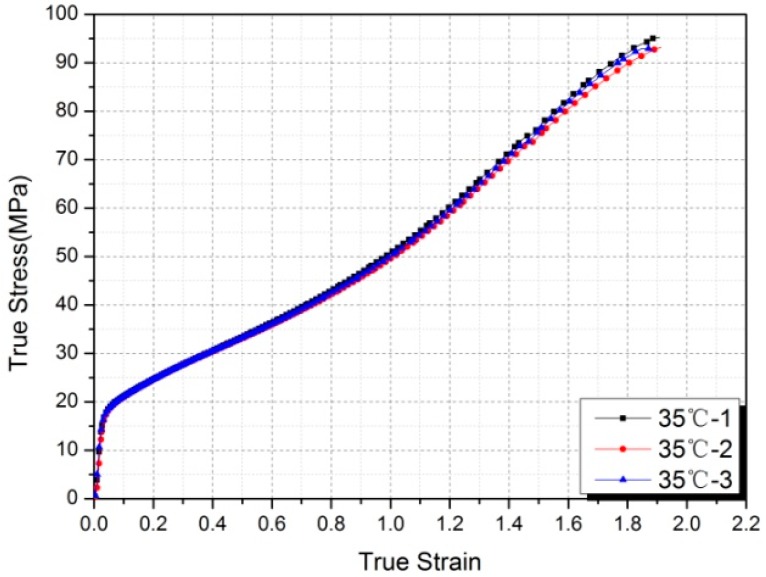
True stress-strain curves of three Al-PTFE samples tested at 35 °C.

Three samples were tested under identical conditions at each temperature and excellent reproducibility between samples was achieved. Taking the experimental results of samples at 35 °C as an example, the stress-strain curves of the three Al-PTFE samples almost overlapped together (Figure 5), which provided confidence that the data obtained in this paper are reliable.

**Figure 6:**
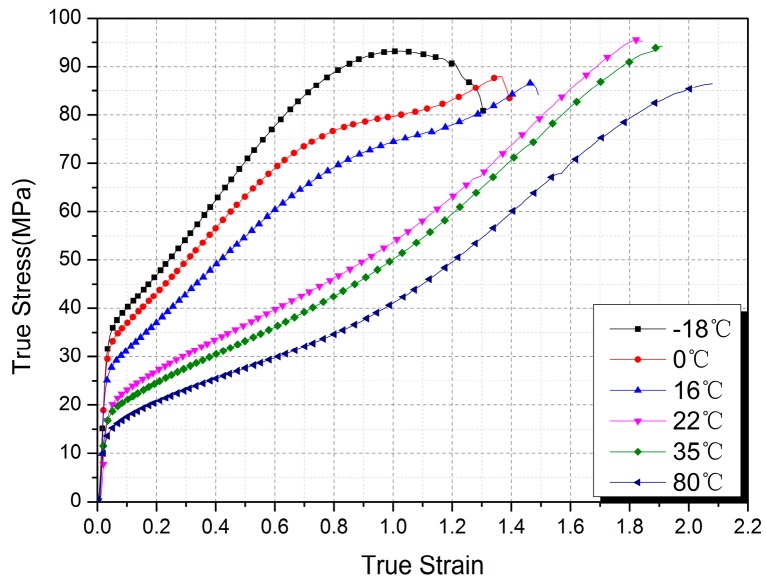
True stress-strain curves of Al-PTFE samples tested at different temperatures.

The true stress-strain curves of Al-PTFE samples tested at different temperatures are shown in Figure 6. The results presented are the average of three tests at each temperature and the standard deviation is less than 1.6 MPa. As expected, the mechanical response showed significant temperature dependence. At low temperatures (−18, 0, and 16 °C), a stress-drop phenomenon was observed before the failure of samples, which was not manifested at high temperatures (22, 35, and 80 °C).

**Figure 7:**
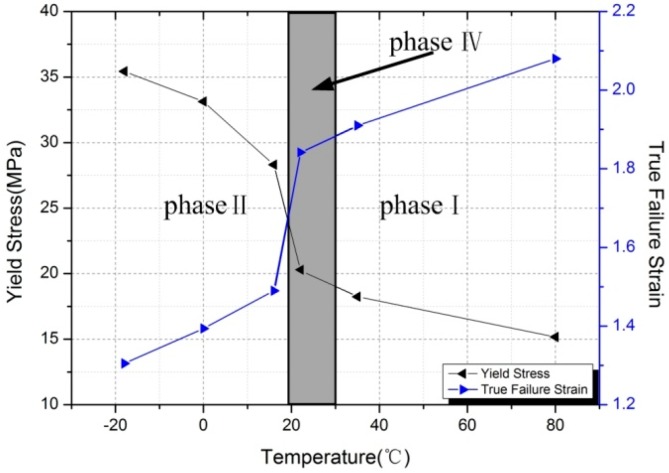
The yield stress and true failure strain of samples as a function of temperature.

**Figure 8:**
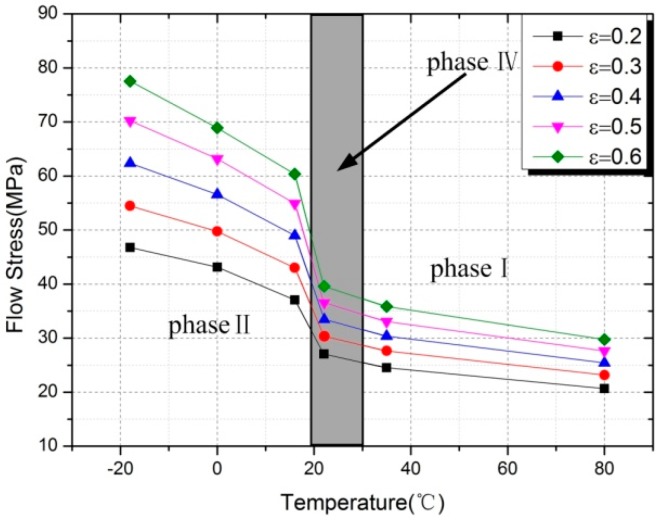
The flow stress at different strains as a function of temperature.

As can be seen from Figure 7, Al-PTFE underwent a gradual transition from brittleness to ductility with increased temperature. The yield stress and true failure strain of samples were strongly temperature-dependent. This was reflected by a 57% decrease of yield stress from 35.43 MPa at −18 °C to 15.17 MPa at 80 °C, and a 59% increase of true failure strain from 1.31 to 2.08 over the same temperature range. Besides, a very unusual change was observed between 16 and 22 °C. Both of the mechanical parameters of Al-PTFE exhibited bilinear temperature dependence, with a high slope below 16 °C and a low slope above 22 °C. In addition to the reduction in yield stress with increased temperature, a similar temperature dependence was also observed in flow stress during plastic deformation. Figure 8 showed the curves of flow stress vs. temperature at different true strains, which are almost identical with the curve of yield stress vs. temperature. At the same true strains, the flow stress of Al-PTFE dropped dramatically at low temperatures (below 16 °C) and then to a lesser degree after an unusual change (between 16 and 22 °C).

Similar relationships between mechanical properties of PTFE and temperatures were reported in previous studies. Speerschneider and Li [23] found that PTFE showed a crystalline size effect and stress drop phenomenon in its flow-stress relations at low temperatures. Brown et al. [24] found that PTFE underwent transitions from brittle-fracture below 19 °C to ductile fracture with large-scale plasticity over 30 °C associated with crystalline phase transformations in tensile tests. Rae et al. [25,26] investigated the properties of PTFE in compression and tension. The results showed that the mechanical properties were strongly affected by strain-rate and temperature. Moreover, the temperature dependence of the Young’s modulus and yield stress both appeared to be bimodal, with a steeper slope below temperature for PTFE in phase II and a second shallower slope above temperature for PTFE in phase I. The research works mentioned above demonstrated that the mechanical properties of Al-PTFE were dominated by PTFE matrix.

### 3.2. Fractography

Because of the complete reaction of Al-PTFE samples at high temperatures (the reaction phenomenon was discussed in detail in Section 3.3), there were no samples left after tests and the fracture mechanism could not be analyzed. To further understand the dependence of mechanical response on temperature, PTFE samples were prepared and compressed under identical conditions and the fracture surfaces of recovered samples were characterized with SEM. Areas of interests were coated with gold to promote electrical conductivity.

**Figure 9:**
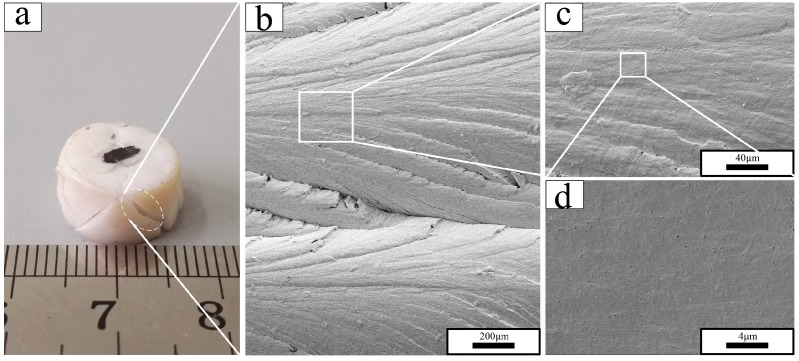
Recovered sample and fracture surface morphology under different magnifications of PTFE tested at −18 °C: (**a**) recovered PTFE sample; (**b**) magnification of 100; (**c**) magnification of 500; (**d**) magnification of 5000.

**Figure 10:**
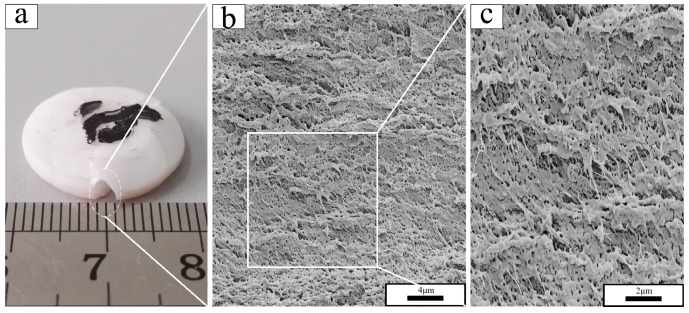
Recovered sample and fracture surface morphology under different magnifications of PTFE tested at 22 °C: (**a**) recovered PTFE sample; (**b**) magnification of 5000; (**c**) magnification of 10,000.

**Figure 11:**
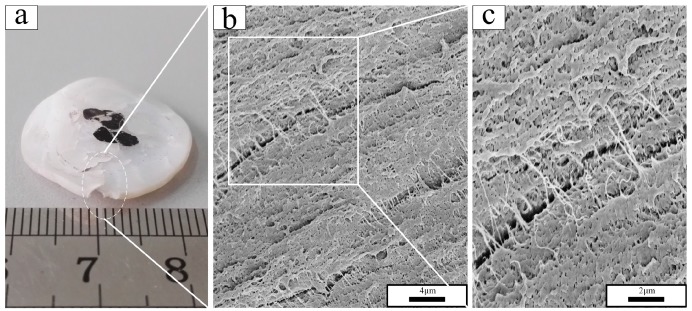
Recovered sample and fracture surface morphology under different magnifications of PTFE tested at 35 °C: (**a**) recovered PTFE sample; (**b**) magnification of 5000; (**c**) magnification of 10,000.

The macroscopic deformation of PTFE at different test temperatures differed markedly (Figure 9a, Figure 10a, and Figure 11a), and a brittle-ductile transition was observed. PTFE in phase II, as represented at −18 °C in Figure 8, exhibited a brittle fracture morphology with a smooth surface (Figure 9c). Fracture propagated radially through the sample, as indicated by linear river markings in Figure 9b. Microvoids were observed on the fracture surface under higher magnification (Figure 9d). Completely different from PTFE in phase II, PTFE in phase IV, as represented at 22 °C in Figure 10, fractured with a high failure strain. The SEM micrographs revealed a rough fracture surface (Figure 10b) and the formation of PTFE fibrils was observed (Figure 10c). The diameter of fibers was as small as ~100 nm. In this sense, they could be termed nanofibrils. Similar to PTFE in phase IV, PTFE in phase I, as represented at 35 °C in Figure 11, exhibited substantial plastic deformation. PTFE fibers could also be seen in Figure 11b,c, in which they became longer and thicker.

The stability of drawing fibrils is primarily determined by temperature and crystalline phase with additional dependence on loading rate and microstructure anisotropy [27]. O’Leary and Geil [28] measured the structure of an individual fibril using electron diffraction. The result shows a large perfect, low molecular weight crystal. Kitamura et al. [29] thought the formation of fibrils is a process of unraveling of the crystalline domains to form an oriented ribbon-like crystalline structure in the direction of loading. Ariawan et al. [30] also suggested that fibrils are oriented amorphous PTFE formed by the unwinding of the crystalline domains, while Brown and Dattelbaum [27] proposed that PTFE fibrils nucleate from a point of stress concentration. Although the formation mechanism and precise structure of fibrils is still not clear, it is believed that the fibrils provide an additional resistance to crack propagation and enhance the ductility of the material. On the one hand, the formation of fibrils is an efficient mechanism to dissipate energy [31,32]; on the other hand, as the fibrils bride the crack surface, they can blunt the crack tip and slow down the propagation of crack [33,34].

### 3.3. Reaction Phenomenon under Quasi-Static Compression

**Figure 12:**
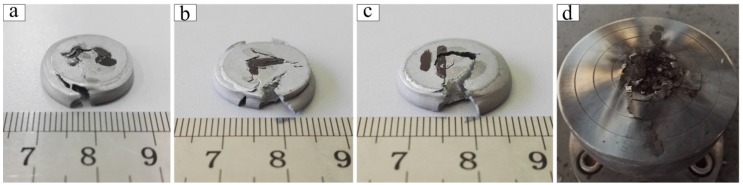
The recovered samples of Al-PTFE after quasi-static compression at different temperatures: (**a**) −18 °C; (**b**) 0°C; (**c**) 16 °C; (**d**) 22, 35, and 80 °C.

Samples tested at 16 °C and below failed with shear crack and no reaction phenomenon was observed (Figure 12a–c), while all of the samples tested at 22 °C and above reacted completely and burned into a pile of black powder (Figure 12d), accompanied by bright flame and explosion sound.

**Figure 13:**
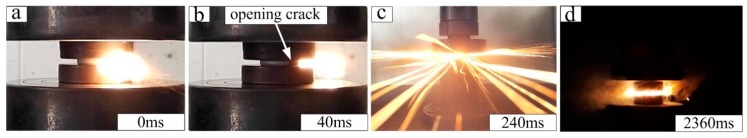
The reaction phenomenon of Al-PTFE samples under quasi-static compression: (**a**) first sign of initiation; (**b**) crack formed along with the initiation; (**c**) violent exothermic reaction; (**d**) the end of reaction.

The reaction process of Al-PTFE samples under quasi-static compression is shown in Figure 13. The reaction originated from the vicinity of the outer surface of the sample (Figure 13a) and the initiation was always accompanied by an opening crack (Figure 13b), which was formed by the transverse tensile stress during compression. Then the reaction spread throughout the sample rapidly (Figure 13c). The whole process lasted about 2360 ms (Figure 13d).

**Figure 14:**
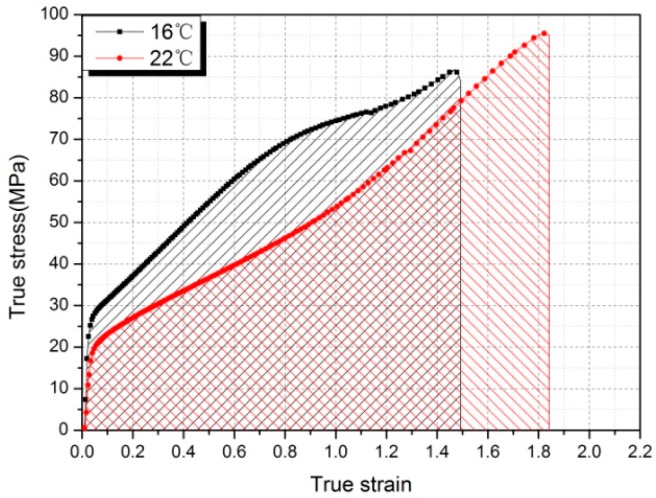
The toughness of Al-PTFE at 16 and 22 °C, respectively.

Feng et al. [35] thought that the reaction phenomenon occurs due to the abrupt energy release along the opening crack in the Al-PTFE sample. The abrupt release of energy absorbed during deformation could cause the temperature to rise high enough to induce a chemical reaction between Al and PTFE at the opening crack tip. High temperatures at the crack tips of polymers which undergo catastrophic failure have been confirmed experimentally by instruments such as thermocouples, infrared detectors, and heat sensitive films [36,37,38]. The magnitude of the rise in temperature is determined by the amount of plastic work and the crack speed [36,37]. In brief, sufficient toughness (the ability of material to absorb energy during deformation, which could be obtained by calculating the area under the stress-strain curve) and an abrupt energy release are two decisive factors for the reaction of the Al-PTFE sample under quasi-static compression. As shown in Figure 14, due to the temperature-induced crystalline phase transformation of the PTFE matrix, the toughness of Al-PTFE showed a 10% increase, from 90.68 J/cm^3^ at 16 °C (phase II) to 99.31 J/cm^3^ at 22 °C (phase IV). Moreover, the true stress-strain curves of Al-PTFE samples at 22 °C and above (phases IV and I) showed no stress-drop phenomenon before failure (Figure 6), which indicated that the energy stored in the samples released instantaneously. Therefore, it is reasonable to speculate that Al-PTFE samples could react under quasi-static compression when subjected to temperatures above 19 °C (crystalline phase transition temperature of PTFE between phases II and IV).

## 4. Conclusions

Quasi-static compression experiments were carried out to investigate the influence of temperature on the mechanical properties and reactive behavior of Al-PTFE. Tests were conducted over a range of temperatures from −18 °C to 80 °C, capturing the three crystalline phases of the PTFE matrix with transitions at 19 and 30 °C. The following conclusions can be drawn from this study:

(1) The mechanical behavior of Al-PTFE is strongly temperature-dependent, and a transition from brittleness to ductility with increased temperature is observed. The yield stress, true failure strain, and flow stress exhibit an unusual change resulting from the temperature-induced crystalline phase transition of the PTFE matrix between phases II and IV.
(2) In the scanning electron micrographs, the PTFE matrix showed two different fracture mechanisms: brittle fracture with a smooth fracture surface in phase II (below 19 °C) and ductile fracture with the formation of PTFE fibrils on the rough fracture surface in phases IV and I (above 19 °C).
(3) This work demonstrates that although Al-PTFE has been considered inert under quasi-static compression, the reaction phenomenon in phases IV and I necessitates the consideration of safety during service at temperatures above 19 °C.
